# Human transcription factors responsive to initial reprogramming predominantly undergo legitimate reprogramming during fibroblast conversion to iPSCs

**DOI:** 10.1038/s41598-020-76705-y

**Published:** 2020-11-12

**Authors:** Ricardo R. Cevallos, Yvonne J. K. Edwards, John M. Parant, Bradley K. Yoder, Kejin Hu

**Affiliations:** 1 Department of Biochemistry and Molecular Genetics, School of Medicine, University of Alabama at Birmingham, Birmingham, AL 35294 USA; 2 Department of Cell Developmental and Integrative Biology, School of Medicine, University of Alabama at Birmingham, Birmingham, AL 35294 USA; 3 Department of Pharmacology and Toxicology, School of Medicine, University of Alabama at Birmingham, Birmingham, AL 35294 USA

**Keywords:** Cell biology, Stem cells

## Abstract

The four transcription factors OCT4, SOX2, KLF4, and MYC (OSKM) together can convert human fibroblasts to induced pluripotent stem cells (iPSCs). It is, however, perplexing that they can do so only for a rare population of the starting cells with a long latency. Transcription factors (TFs) define identities of both the starting fibroblasts and the end product, iPSCs, and are also of paramount importance for the reprogramming process. It is critical to upregulate or activate the iPSC-enriched TFs while downregulate or silence the fibroblast-enriched TFs. This report explores the initial TF responses to OSKM as the molecular underpinnings for both the potency aspects and the limitation sides of the OSKM reprogramming. The authors first defined the TF reprogramome, i.e., the full complement of TFs to be reprogrammed. Most TFs were resistant to OSKM reprogramming at the initial stages, an observation consistent with the inefficiency and long latency of iPSC reprogramming. Surprisingly, the current analyses also revealed that most of the TFs (at least 83 genes) that did respond to OSKM induction underwent legitimate reprogramming. The initial legitimate transcriptional responses of TFs to OSKM reprogramming were also observed in the reprogramming fibroblasts from a different individual. Such early biased legitimate reprogramming of the responsive TFs aligns well with the robustness aspect of the otherwise inefficient and stochastic OSKM reprogramming.

## Introduction

OCT4, SOX2, KLF4, and MYC (collectively OSKM) can convert human fibroblasts into induced pluripotent stem cells (iPSCs), which are the man-made version of embryonic stem cells (ESCs)^1–3^. Although powerful and revolutionary, iPSC reprogramming is very inefficient, slow and stochastic^4,5^. Only a rare population can traverse the reprogramming threshold and reach the state of pluripotency. The molecular underpinnings underlying the potency as well as the tremendous limitations of iPSC reprogramming are poorly understood. Although factor reprogramming is technically more straightforward than the traditional reprogramming by the natural reprogramming vehicle oocyte using somatic cell nuclear transfer (SCNT)^6^, it is still very challenging to dissect the molecular mechanisms of iPSC reprogramming since 99% of the original reprogramming starting cells do not go in the direction towards pluripotency and the collected data from those reprogramming cells may largely represent noises. Previous research reported the genome-wide transcriptional responses to the OSKM reprogramming factors, and assumed implicitly that all of those responses are positive^7,8^. This is logically not appropriate since only less than 1% of the cells undergo authentic pluripotency reprogramming. To address this limitation in the previous research, we recently developed the concept of reprogramome, which is the full complement of genes that should be reprogrammed^9^. Based on the concept of reprogramome, we further developed another concept of reprogramming legitimacy, and these new concepts allow logical evaluations of an early transcriptional response of a gene to the reprogramming factors^10^.

Each cell type has a defined and specific transcriptome. The transcriptome is governed by a defined set of transcription factors. iPSC generation, in essence, is a process of transcriptional reprogramming^2^. To convert fibroblasts into iPSCs, it is essential to erase the fibroblast transcriptional program, and at the same time to establish the pluripotency transcriptional program. Among the transcriptional reprogramming of the entire reprogramome, the most critical process should be to erase the fibroblast transcription factor (TF) system, and at the same time to establish the pluripotent TF network. It is not clear what the full differences in the TF networks are between the starting fibroblasts and the endpoint iPSCs. Our previous analyses of reprogramming legitimacy were general in nature, and did not specifically examine transcriptional responses of the transcriptional factors to OSKM reprogramming.

Realizing the critical roles of TFs in defining cellular identities and cellular reprogramming, this research employed our new concepts of reprogramome and reprogramming legitimacy to evaluate specifically the early responses of human TFs to the OSKM reprogramming. The authors first defined the TF differences, that is, the set of transcription factors that should be reprogrammed, i.e., the TF reprogramome. Specifically, the repertoire of fibroblast-enriched and specific transcriptional factors, the TF downreprogramome, was defined. Similarly, the TF upreprogramome was defined. With the defined TF down- and up-reprogramomes, the reprogramming legitimacy was then evaluated. In agreement with the extremely low efficiency of reprogramming, we found that most members of the TF reprogramome were not responsive to OSKM reprogramming. Surprisingly, we also found that among the TFs that did respond to OSKM reprogramming, most of the transcriptional responses of TFs were legitimate in the context of reprogramming although a small portion of aberrant and unwanted reprogramming were also observed among the responsive TFs. The legitimate reprogramming of the majority of the responsive transcription factors to the early OSKM induction aligns well with the potency aspect of OSKM reprogramming, but at the same time, resistance of some transcription factors to OSKM induction along with some aberrant and unwanted TF reprogramming may underlie the low efficiency, long latency, and stochastic nature of iPSC reprogramming.

## Results

### Defining the set of transcriptional factors to be reprogrammed, the TF reprogramome

**Figure 1:**
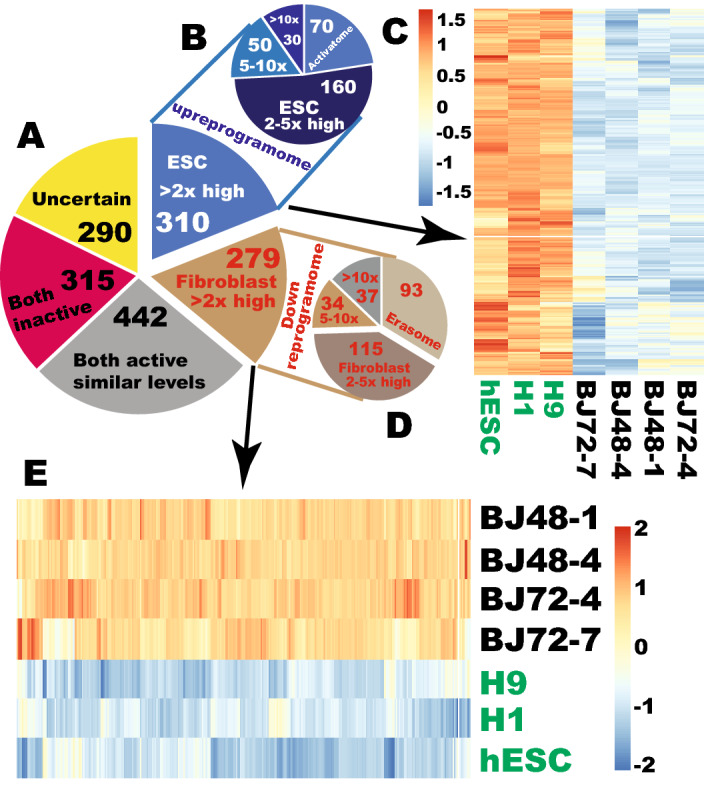
Profiling reprogramming of human transcription factors for fibroblast-to-iPSC conversion. (**A**) Classification of differential expressions for the entire human transcription factor (TF) repertoire. (**B**) Number of TF genes in different degrees of enrichment in human embryonic stem cells (ESCs) as compared to fibroblasts. (**C**) A heat map showing enrichment of 310 human TF genes in hESCs. (**D**) Number of TF genes in different degrees of enrichment in human fibroblasts as compared to hESCs. (**E)** A heat map showing enrichment of 279 human TF genes in human fibroblasts compared with that in hESCs.

Transcription factors (TF) are critical in defining any cell type^11–13^. Human pluripotent stem cells (PSCs) should have a defined set of TFs, so do the starting cells for iPSC reprogramming, fibroblasts. In order to convert human fibroblasts to iPSCs, it is of paramount importance to reprogram the TFs to the expression levels of the pluripotent state from that of fibroblasts. It is not clear what the full TF differences are between the pluripotent cells and the starting somatic fibroblasts, i.e., the TF reprogramome. In order to find out the TF transcriptional differences between PSCs and fibroblasts, this study compared the expression of the entire set of human TFs based on RNA-seq data we recently published^9,10^. Several groups attempted to define the repertoire of human TFs^14–16^. The latest revised version by Lambert et al. was used in this study^17^. The RNA-seq data were extracted for the Lambert set of 1639 human TFs, but the current report concerns with 1636 TFs only because *ZNF788* is a pseudogene, and *DUX1* and *DUX3* were not annotated in the Ensembl database (Table S1). Of the 1636 TFs, 315 were not expressed in both ESCs and fibroblasts, while 442 TFs were expressed in both cell types at similar levels (Fig. 1A). Two hundred and seventy-nine (279) TFs were enriched by at least 2-fold (q < 0.01) in fibroblasts compared to human ESCs, and they constitute the TF downreprogramome (Table S2, and Fig. 1A,D,E, and Fig. S1). There are 110 zinc finger, 18 HOX, 11 forkhead box, and 7 T-box TF genes in the downreprogramome. Within the TF downreprogramome, 93 TFs were expressed in fibroblasts only, constituting the TF erasome (Table S3, Fig. 1D). Of note, all of the 18 HOX TF genes are in the erasome, and there are 32 zinc finger TF genes in the erasome. Excluding the erasome, additional 71 TFs of the downreprogramome were highly enriched (by > 5-fold) (Fig. 1D). In the entire downreprogramome of 279 TFs, 217 were enriched by at least 3-fold.

Three hundred and ten (310) TFs are enriched in hESCs by at least 2-fold, and constitute the TF upreprogramome (Table S4, Fig. 1A–C, and Fig. S2). As expected, the established pluripotent TFs are in the upreprogramome including *POU5F1*, *NANOG*, *SOX2*, *ZFP42*, *ZSCAN10*, *FOXD3*, *PRMD14*, *ZIC3*, *SALL4*, and others. Interestingly, there are 198 zinc finger TF genes of different types (genes with the designations of ZNF, ZFP, ZIC, ZSCAN, GATA, KLF, SALL, ZBTB, and others) in the upreprogramome. Therefore, zinc finger TFs represent 63.9% of the TF upreprogramome. In contrast to the downreprogramome, there is not a single HOX gene in the upreprogramome. In fact, all the 39 HOX genes of human genome are silenced in hESCs. The upreprogramome contains 8 SOX, 4 POU, and 4 SALL genes but the downreprogramome includes no SALL and SOX genes and only one POU gene. Within the TF upreprogramome, 70 TFs were expressed in hESCs only, constituting the TF activatome (Table S5, Fig. 1B). Excluding the activatome, 80 additional TFs are highly enriched in hESCs (by > 5-fold) (Fig. 1B). There were 28 zinc finger TFs in the activatome. Two hundred and ninety of the human TFs could not be classified into the above categories because they fell into the marginal areas based on the selection criteria used here (Fig. 1A). Combining the TF downreprogramome and upreprogramome, the TF reprogramome includes 589 TFs that should be reprogrammed by at least 2-fold. Interestingly, the downreprogramome is characterized by HOX genes while the upreprogramome is featured by SOX, SALL, and POU TFs, and is dominated by zinc finger TFs of different types.

GO analyses of the upreprogramome and downreprogramome resulted in very different pictures. The majority of the pluripotent TF GO terms were generic while the fibroblast ones were very promiscuous. At the FDR < 0.01 level, the TF downreprogramome was overrepresented by 428 different GO terms (Table S6) while the TF upreprogramome was overrepresented by 103 GO terms only (Table S7). Of these 103 pluripotent TF GO terms, 90 were shared with that of fibroblasts. Among the 13 unique GO terms for ESCs were “chromatin organization” (26 genes), “stem cell population maintenance” (15), “regulation of cell cycle arrest” (9), and “maintenance of cell number” (15) (Fig. S3). As expected, ESC TFs were overrepresented in “reactome pathways” analyses by the GO term of “transcriptional regulation of pluripotent stem cells” (11 genes), but “generic transcription pathway” (116) and “RNA polymerase II transcription” (119) were also overrepresented. Among these GO terms unique to fibroblasts were “response to chemical” (99 genes), “response to stress” (79), “regulation of programmed cell death” (57), “limb development” (26), “muscle tissue development” (21), “brain development” (27), and “response to mechanical stimulus” (12) (Fig. S4). One interesting GO term in “reactome pathway” analyses of fibroblast-enriched genes was “transcriptional regulation of white adipocyte differentiation” (11 genes). In sum, the enriched GO terms for fibroblast TFs appear to be more tissue-specific and promiscuous, and those for pluripotent TFs are more generic and stem cell-related.

### A portion of the TF reprogramome are resistant to reprogramming

**Figure 2:**
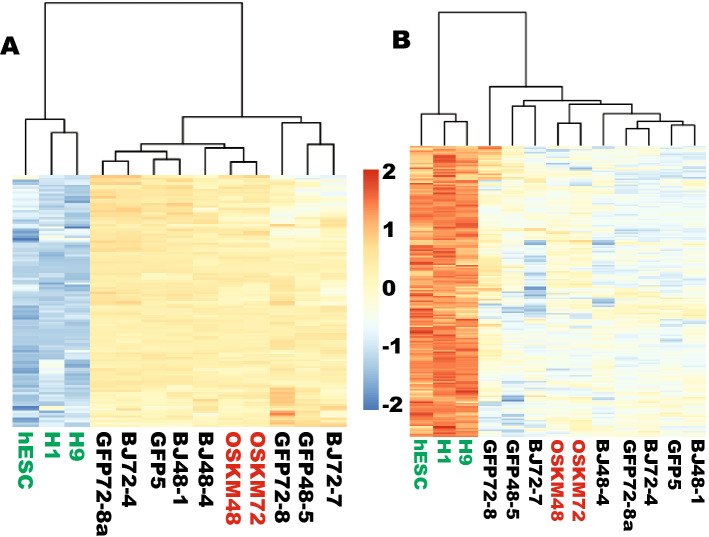
A group of transcription factors in the TF reprogramome is resistant to OSKM reprogramming at the early stages. (**A**) A heat map showing that 108 fibroblast-enriched TFs were irresponsive to OSKM reprogramming at the initial stages. (**B**) A heat map showing that 188 PSC-enriched TFs were irresponsive to OSKM reprogramming at the initial stages. OSKM, OCT4, SOX2, KLF4 and MYC. 48 and 72 after OSKM indicate time for RNA harvest post OSKM transduction of fibroblasts. OSKM reprogramming cells are highlighted in red. Human embryonic stem cells (ESCs) are highlighted in green.

Yamanaka reprogramming is very inefficient, slow and stochastic. Previously, we reported that 953 genes are resistant to iPSC reprogramming at the initial stages^10^. We hypothesized that a portion of transcription factors in the reprogramome is among those genes irresponsive to OSKM reprogramming considering the low efficiency and long latency of iPSC reprogramming. To test this, the TF reprogramome was examined for the reprogramming statuses of its member genes upon OSKM reprogramming. Indeed, 108 out of the 279 fibroblast-enriched TFs were irresponsive at both time points examined (Figs. 2A, Fig. S5, and Table S8), while 188 out of the 310 PSC-enriched TFs demonstrated no significant transcriptional changes by OSKM reprogramming for 72 h (Figs. 2B, S6, and Table S9). Clustering analyses indicated that these irresponsive TFs remained similar to those in the starting naïve fibroblasts and in the fibroblasts transduced with GFP. In the upreprogramome, 124 zinc finger TFs were irresponsive to OSKM reprogramming including 4 ZSCAN zinc finger genes (*ZSCAN2*, *ZSCAN10*, *ZSCAN16*, and *ZSCAN31*). In the downreprogramome, 13 HOX genes were resistant to OSKM reprogramming.

As expected, 7 ESC-enriched genes with the “reactome pathway” GO term of “transcriptional regulation of pluripotent stem cells” were among the TF genes resistant to reprogramming. These include *NANOG*, *LIN28A*, *ZSCAN10*, *FOXD3*, *PRDM14*, *ZIC3*, and *HIF3A*. Interestingly, 66 ESC-enriched genes of the “reactome pathway” GO term of “generic transcription pathway” were also resistant to OSKM reprogramming.

### Transcriptional responses of transcription factors to OSKM induction

Next, we defined the set of TF genes significantly upregulated (TF upregulatome of OSKM) and downregulated by OSKM (TF downregulatome of OSKM). In the analyses of transcriptional impact of OSKM on TFs, we used two reference conditions: naïve fibroblasts and the fibroblasts transduced with GFP viruses. The use of GFP control can remove the transcriptional impact by viruses per se. We found that OSKM upregulated 53 TFs by at least 2-fold (q < 0.01) compared to both the naïve fibroblasts and the fibroblasts transduced with the GFP viruses (Fig. S7, and Table S10). As expected, the four reprogramming factors *OCT4* (*POU5F1*), *SOX2*, *KLF4*, and *MYC* were among this list. Therefore, only 49 TFs were significantly upregulated by at least 2-fold at the initial stages. The fold upregulation ranged from 2- to around 17-fold and up to de novo activation of 12 TF genes. On the other hand, 70 TFs were downregulated by OSKM by at least 2-fold (q < 0.01) compared to the naïve fibroblasts and the fibroblasts transduced with GFP viruses (Fig. S8 and Table S11). The fold downregulation ranged from 2- to 14-fold, and up to silencing of 5 TF genes.

### Most of the downregulation of TFs by OSKM is legitimate reprogramming

**Figure 3:**
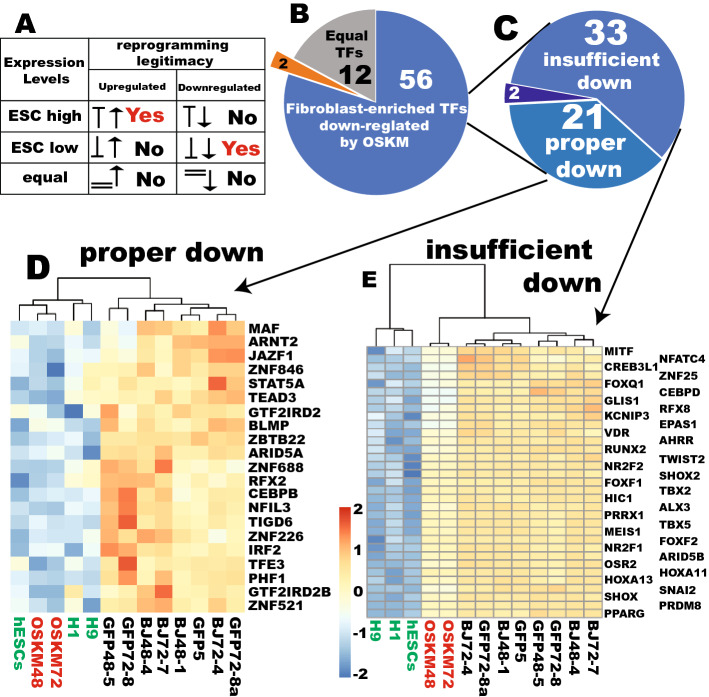
The majority of the initial TF downregulation by OSKM is legitimate reprogramming. (**A**) Logic for evaluation of reprogramming legitimacy. (**B**) Classification of the 70 TFs downregulated by OSKM into different expression statuses in PSCs and the reprogramming starting fibroblasts. The orange sector indicates that 2 TFs are expressed at least 2-fold higher in ESCs. (**C**) Further characterization of reprogramming status for the 56 fibroblast-enriched genes downregulated by OSKM. (**D**) A heat map showing proper downreprogramming of the 21 fibroblast-enriched TFs. (**E**) A heat map showing insufficient downreprogramming for the significantly downregulated 33 fibroblast-enriched TFs by OSKM.

The legitimacy of a transcriptional change of a TF induced by OSKM should be evaluated by the relative expression levels of individual TFs in PSCs to that in fibroblasts^10^ (Fig. 3A). Upregulation of a gene is legitimate if its expression is higher in PSCs, while it is not when its expression is lower in PSCs. On the other hand, downregulation of a gene is legitimate if its expression is lower in PSCs while it is not when its expression is higher in PSCs. If the expression level of a gene is similar in both cell types both up- and down-regulations by the OSKM reprogramming factors are illegitimate, which constitute aberrant reprogramming^10^.

Using this logic, we evaluated the 70 TFs downregulated by OSKM for their reprogramming legitimacy. Surprisingly, only one TFs was enriched in ESCs by at least 2-fold and 49 were enriched in fibroblasts by at least 2-fold (Table S12). Because of this biased enrichment of the downregulated TFs for fibroblasts, the criteria were then loosened to a significance level of q < 0.05 for significant differences at any level. Fifty six out of the 70 genes downregulated by OSKM were expressed significantly higher in fibroblasts by at least 1.46-fold, indicating legitimate downreprogramming of these 56 TFs (Fig. 3B). A scrutiny of the 56 genes indicated that 21 were properly reprogrammed and became clustered with ESCs and away from fibroblasts and the fibroblasts transduced with GFP viruses (Figs. 3C,D, S9). Thirty-three of those 56 TFs were downregulated significantly towards the pluripotency levels although the downreprogramming is insufficient (Figs. 3C,E, S10), indicating a positive drive to the pluripotency states with some deficiency. In summary, 80% of the downregulated TFs by OSKM underwent legitimate downreprogramming.

### Most of the upregulation of TFs by OSKM is legitimate reprogramming

**Figure 4:**
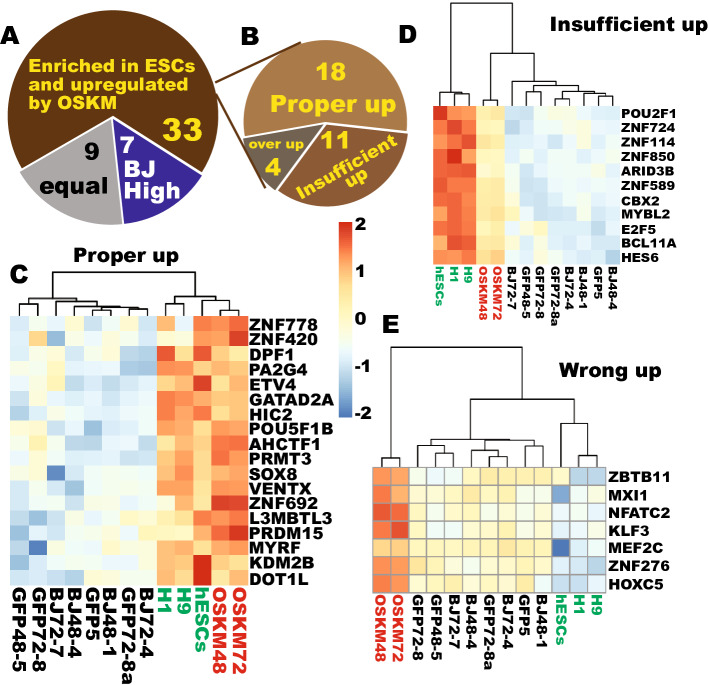
Most of the initial TF upregulation by OSKM is legitimate reprogramming. (**A**) Classification of the 49 TFs upregulatd by OSKM into different expression statuses in PSCs and the reprogramming starting fibroblasts. (**B**) Further reprogramming characterization of the 33 ESC-enriched genes upregulated by OSKM. (**C**) A heat map showing proper upreprogramming of 18 ESC-enriched TFs. (**D**) A heat map showing insufficient upreprogramming for the 11 ESC-enriched TFs significantly upregulated by OSKM. (**E**) A heat map showing wrong upreprogramming by OSKM for the 7 fibroblast-enriched TFs.

Next, we evaluated the reprogramming legitimacy of the 49 upregulated TFs. Of note, only three of them were expressed higher in fibroblast by at least 2-fold. Because biased enrichment was observed again, the criteria were then loosened to the significance level of q < 0.05 at any difference level as done for the 70 TFs downregulated by OSKM. New criteria resulted in only 7 TFs that were enriched in fibroblasts by at least 1.44-fold (Fig. 4A). There was no difference in expression for nine of them, of which three were not expressed in both cell types. Impressively, 33 out of the 49 upregulated TFs were enriched in PSCs by at least 1.37-fold (25 TFs by at least 2-fold and 7 by 1.5- to 2-fold) (Fig. 4A), indicating that 67.5% of the upregulation is legitimate reprogramming. Detailed examination showed that 18 of the 33 PSC-enriched TFs were properly upreprogrammed and became clustered with the PSCs and away from the starting fibroblasts and the reprogramming GFP controls (Figs. 4B,C, and Fig. S11), while 11 of them were insufficiently upreprogrammed and remained clustered with the starting cells (Fig. 4B,D, and Fig. S12). However, 7 of the fibroblast-enriched genes upregulated by OSKM were wrongly upreprogrammed and became a separate independent group in clustering analyses (Fig. 4A,E). Of the 9 TFs with similar expression in both cell types, three can be considered as unwanted activation because they are not expressed in both cell types while four are unwanted upreprogramming (data not shown). In summary, legitimate reprogramming is predominant among the 49 upregulated TFs (67.5%).

### Independent human fibroblasts from a different individual have a similar TF reprogramome

Fibroblasts are heterogeneous. Although the human fibroblast BJ used above has been widely employed in research of pluripotency reprogramming^1–3,18^, we investigated whether an independent fibroblast from a different individual has similar TF reprogramome. To this end, we used another model human fibroblast line also used in reprogramming study previously, CRL-2097 (denoted as CRL hereafter)^19,20^. We also used a different RNA-seq technology DNA nanoball sequencing (DNB-seq) because it is much cheaper than the Illumina technology we used for the above data. Our additional data indicated that the CRL fibroblast has a very similar TF upreprogramome to that of BJ (Supplementary Fig. S13, Supplementary Table S13). In short, for the new fibroblast CRL, 350 TFs were expressed significantly higher in ESCs, of which 263 TFs were shared with BJ (Fig. S13A,B). Out of the remaining 87 TFs not shared by BJ, 59 were still expressed significantly higher in ESCs than in the BJ when the sorting criteria were loosened (p < 0.05 with significant differences at any levels) (Fig. S13C). At the same time, out of the 47 ESC-enriched TFs in the BJ list but not in the CRL list, 28 were still enriched in ESCs compared to CRL when the selection criteria were loosened (Fig. S13D). Notably, only in one rare case we saw conflicting results between the two fibroblasts. *DBP* expression was significantly higher (2.1 ×) in ESCs compared to one fibroblast (CRL) but significantly lower (− 1.6 ×) in ESCs compared to another fibroblast (BJ).

Similarly, the majority of the TF down-reprogramomes were shared by the two fibroblasts (Supplementary Fig. S14, and Supplementary Table S14).

### The initial TF responses to Yamanaka factors in an independent human fibroblast are predominantly legitimate reprogramming

**Figure 5:**
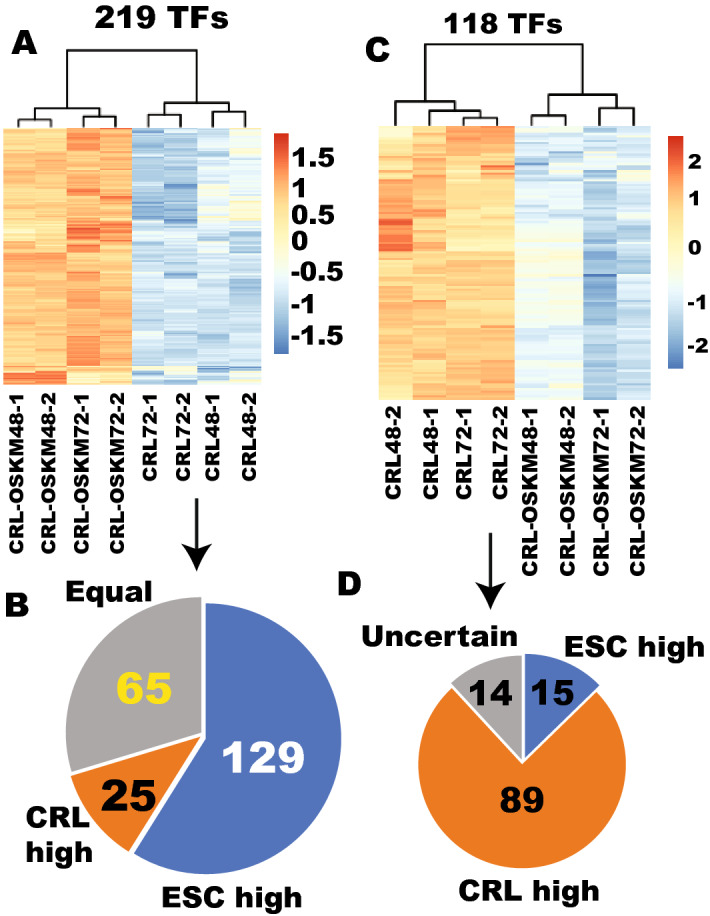
The TFs that were responsive to OSKM reprogramming in an independent fibroblast (CRL) are predominantly legitimate. (**A**) 219 TFs were significantly upregulated by at least 2-fold by OSKM (q < 0.01, fibroblast = 4, OSKM = 4). (**B**) 129 out of the 219 OSKM-upregulated TFs are enriched in ESCs by at least 2-fold. (**C**) 118 TFs were significantly downregulated by OSKM in CRL by at least 2-fold (q < 0.01, fibroblast = 4, OSKM = 4). (**D**) 89 out of the 118 OSKM-downregulated TFs were enriched in fibroblasts by at least 2-fold.

Next, we investigated whether the initial legitimate reprogramming of transcription factors can be observed in an independent fibroblast cell line. For this purpose, we sequenced RNA from the fibroblast CRL undergoing early reprogramming. As in BJ cells, OSKM were all overexpressed well in CRL cells in all samples (Supplementary Fig. S15). OSKM upregulated 219 TF at both 48 and 72 h post factor transduction (Fig. 5A and Supplementary Table S15). As seen with the BJ cells, these upregulated TF are predominantly ESC-enriched (129 out of 219) (Fig. 5B). Classification of “insufficient up”, “proper up” and “over up” eliminate some genes with legitimate reprogramming because of the stringent sorting criteria applied. In fact, these 129 TFs can be considered as legitimate reprogramming (insufficient up, proper up and over up) (Fig. 3A) since overexpression of pluripotency factors might be beneficial to reprogramming as we have seen with the OCT4 and SOX2 reprogramming factors. We also examined the situation of these 129 TFs in BJ cells. None of these 129 TFs have significantly higher expression in BJ cells than in ESCs and the majority of them (116) are expressed significantly higher in ESCs than in BJ (Supplementary Fig. S15 and data not shown). None of these 129 genes was significantly downregulated by OSKM in BJ cells, but the majority of them (104) were significantly upregulated by OSKM in BJ cells (Supplementary Fig. S16 and data not shown). These 129 TFs of legitimate reprogramming in CRL were also clustered with ESCs at 96 h of OSKM reprogramming of BJ cells, indicating their legitimate reprogramming in BJ cells as well (Supplementary Fig. S16). However, 65 TFs were upregulated by OSKM in CRL cells when they should not be, and 25 TF were upregulated when they should be downregulated (Supplementary Table S15). Nevertheless, the majority (59%) underwent legitimate upreprogramming and were largely conserved between the two fibroblast types.

A total of 118 TFs were downregulated by OSKM in the CRL cells at both 48 and 72 h (Supplementary Table S16). Out of the 118 genes, 89 were enriched by at least 2-fold in the fibroblast CRL, indicating legitimate downreprogramming (Fig. 5C,D). Importantly, none of these 89 TFs exhibited significantly higher expression in ESCs than in the other fibroblast BJ, and the majority of them (79 TFs) displayed significantly higher expression in BJ than in ESCs (Supplementary Fig. S17 and data not shown). Furthermore, none of the 89 genes was significantly upregulated and the majority (71 TFs) of those were significantly downregulated in BJ cells by OSKM at 96 h of reprogramming. Like in CRL, these 89 genes became clustered with ESCs in the reprogramming CRL cells at 96 h (Supplementary Fig. S17), indicating conserved legitimate downreprogramming between the two different fibroblasts.

## Discussions

OSKM can convert a rare population of fibroblasts into the pluripotent state with an extended long latency^4,5^. This is in sheer contrast to the oocyte reprogramming, which is authentic and fast^6^. OSKM cannot activate the master transcriptional network of pluripotency directly. The key pluripotent factors, OCT4, SOX2, NANOG and others, are activated very late in the reprogramming process. Previous efforts have tried to identify molecular events underlying OSKM reprogramming of fibroblasts into pluripotency. However, those researches ignored the fact that 99% of the cells do not go in the direction towards pluripotency and represent the noise signals of the data. Those authors implicitly treated all the transcriptional responses to OSKM induction as positive reprogramming. To mitigate this limitation, the authors developed the concepts of reprogramome and reprogramming legitimacy^9,10^. Using these concepts, a transcriptional response to the OSKM reprogramming can be evaluated as positive, negative (aberrant reprogramming), or irresponsive i.e., legitimate, illegitimate, and no responses, respectively. In previous reports, the authors evaluated the transcriptional responses of all human genes without specific examination of the transcription factors. In this report, the authors evaluated the reprogramming legitimacy of the transcriptional responses of the entire set of human transcription factors to OSKM reprogramming at the initial stages (48, 72, and 96 h). In agreement with the inefficiency, long latency, and stochastic nature of OSKM reprogramming, it was found here that the majority of human transcription factors (296 TFs) were irresponsive to OSKM induction. This report also identified some transcription factors that underwent aberrant reprogramming such as wrong and unwanted reprogramming. These data provide molecular interpretation for the inefficiency and stochastic nature of OSKM reprogramming.

When we specifically analyzed the reprogramming legitimacy of TFs in this report, a surprising discovery is that the majority of transcription factors, which did respond to OSKM induction, underwent legitimate reprogramming. This phenomenon was also observed in an independent human fibroblast cell line from a different individual. The population of transcription factors undergoing legitimate reprogramming is not small. 18 PSC-enriched TFs were properly upreprogrammed to the levels found in PSCs, while 21 somatic TFs were properly downreprogrammed to the levels of pluripotency. Additionally, 11 PSC-enriched TFs were significantly albeit insufficiently reprogrammed towards the pluripotent levels while 33 somatic TFs were significantly downregulated albeit insufficiently downreprogrammed towards the pluripotent levels. These observations may under-estimate the number of legitimate TF reprogramming since classification of legitimate reprogramming into proper, insufficient and over reprogramming usually eliminate some genes undergoing legitimate reprogramming as outlined in Fig. 3A. In fact, we observed many more legitimate TF reprogramming simply using the rationale in Fig. 3A (Fig. 5).

Transcription factors are critical in defining the transcriptional programs and identities of any cell type^11–13^. TFs are also critical in cellular reprogramming. In fact, all the four conventional pluripotency reprogramming factors are transcription factors^21^. Lineage-specific transcription factors can reprogram fibroblasts into the corresponding functional somatic cells^22–24^. Here, the legitimate reprogramming of a large set of transcription factors at the initial stages provides molecular underpinnings for the ability of OSKM to push some reprogramming fibroblasts to the pluripotent state. At the same time, the inability of OSKM to incite the required transcriptional changes of the transcription factors in the TF reprogramome at the early stages explains in part why OSKM is very inefficient, slow and stochastic.

## Methods

### Cell lines and cultures

The NIH-registered human embryonic stem cell (ESC) lines H1 (WiCell, Madison, WI) and H9 (WiCell, Madison, WI) were cultured in the chemically defined media as described before^2,3,9,10^. Briefly, hESCs were cultured on Matrigel-coated vessels with the E8 media^25^, and passaged using the EDTA-mediated dissociation when they reach 80% confluency.

Human primary fibroblasts (BJ, ATCC, CRL-2522, and CCD-1079Sk, ATCC, CRL-2097) with normal karyotypes were cultured in the fibroblast medium: Dulbecco’s Modified Eagle Medium (DMEM) with high glucose, supplemented with 10% heat-inactivated fetal bovine serum, 0.1 mM 2-mercaptoethanol, 1 × penicillin–streptomycin, 0.1 mM Minimum Essential Medium Non-Essential Amino Acids, and 4 ng/mL human FGF2.

### Lentivirus vector production

Lentivirus vectors were generated using the PEI-mediated transfection of Lenti-X 293T cells (Takara, Cat. 632180) by the reprogramming plasmids. Briefly, 2 × 10^7^ Lenti-X 293T cells were seeded into one 150-mm dish and cultured in expansion medium: DMEM-F12 (Gibco, Cat. 12400-024) supplemented with 10% FBS (Gibco, Cat. 10437-028). Twenty-four hours post seeding and at least 2 h before transfection, the spent medium was replaced with 24 mL of fresh expansion medium. Mix the envelope, packaging and transfer plasmids at a ratio of 1:3:4 (total amount of 60 µg of plasmids) in 3 mL of DMEM-F12 medium, and then mix the plasmid solution with 3 mL of “PEI solution” containing 60 µg/mL of Polyethylenimine “Max” (PEI, Polysciences Inc., Cat. 24765-2). Incubate the 6 ml of transfection mix for 15 min at room temperature, and then add the resulting DNA complex dropwise into the cell cultures. The cultures were incubated for 16 h at 37 °C, 5% CO_2_. After the 16-h transfection, the transfection medium was replaced carefully with 20 ml of fresh complete expansion medium, and the cells were incubated for additional 72 h. Medium containing lentiviral particles was then harvested and filtered using Stericup PVDF membrane filters of 0.45-µm pore size (Millipore). Filtered virus-containing supernatant was concentrated by addition of 50% PEG6000 stock solution into the viruses to a final concentration of 8.5% PEG6000 and 4 M NaCl stock to a final concentration of 0.4 M NaCl. Precipitate the viruses by incubating at 4 °C for 3 h with gentle mixing every 30 min. Collect the precipitated viruses by centrifugation at 4500 × g for 45 min. Lentiviral pellets were re-suspended into 150 µL sterile PBS, and immediately stored in small aliquots at -80 °C. Titration of lentiviral preparations was performed by transducing HeLa cells (ATCC, CCL-2) and analyzing the GFP expression using flow cytometry (Fortessa Flow Cytometer, BD) 72 h post transduction.

### Initial reprogramming

Our lentiviral reprogramming constructs were reported before and have been deposited in Addgene^3^ (pLVH-EF1a-GFP-P2A-OCT4, Addgene #, 130692; pLVH-EF1a-GFP-P2A-SOX2, Addgene #, 130693; pLVH-EF1a-GFP-P2A-KLF4, Addgene #, 130694; pLVH-EF1a-GFP-P2A-MYC, Addgene #, 130695). All these constructs have GFP co-expression for easy estimation of viral titers, as well as transfection and transduction efficiency. The reprogramming procedure was basically as reported before^3,26^ except for that reprogramming was stopped at the indicated time points when RNA was extracted. Human fibroblasts were transduced with the concentrated lentiviruses of the four Yamanaka reprogramming factors at the optimized multiplicity of infection (MOI) in the authors’ lab (OCT4, 8; SOX2, 5; KLF4, 5; MYC, 3) in the presence of polybrene at 4 μg/mL. At 12 h post transduction, the residual viruses and viral debris were removed by a medium change. The transduced cells were cultured in fibroblast medium until RNA harvest, which were 48, 72, and 96 h post transduction. We consistently reach > 90% of transduction efficiency over the years using these constructs as judged by GFP expression and flow cytometry^3^ (Supplementary Fig. S15). Efficient overexpression of all four reprogramming factors in all of our nine reprogramming RNA-seq samples was indicated by the elevated normalized read counts of the transgenes in each sample (Supplementary Fig. S15)^10^.

### RNA preparation for RNA-seq

RNA was first isolated from the monolayer cultures at the desired time points of treatments using TRIzol Reagent (Invitrogen, Cat. 15596026). After precipitation with 70% ethanol, RNA pellet was resuspended in 80 μL of 1 × DNAse digestion buffer supplemented with 5 units of DNAseI (Thermo Fisher Scientific, Cat. AM2222). The reaction was incubated for 10 min at RT. The reaction was stopped and cleared using Quick-RNA Miniprep columns (Zymo Research, Cat. R1054). The RNA was eluted in 50 µL nuclease-free water, and stored at − 80 °C. RNA concentration was determined using Nanodrop spectrophotometer (Thermo Fisher Scientific). RNA quality was also assessed by agarose-gel electrophoresis and high-resolution electrophoresis.

### RNA-seq and bioinformatics

The 19 samples of DNB-seq RNA-sequencing comprise four groups with four biological replicates each and a fifth group with three biological replicates (Supplementary Table S17). Paired-end 100 bp reads were sequenced utilizing the DNBSEQ-G400 sequencing instrument at BGI. Pre-alignment quality assessments of the raw fastq sequences were carried out using FastQC (version 0.11.7)^27^. The number of paired-end reads for the 19 samples ranged from 26.4 to 31.9 M (Table S17). The fastq sequences did not require adapter removal, trimming, or filtering. The raw fastq sequences were aligned to the human hg38 reference genome (GenBank assembly accession: GCA_000001405.28). The alignments were carried out using STAR (version 2.7.1a)^28^ with the default parameters. Post-alignment quality assessments were carried out with RSeQC (version 2.6.3)^29^ and MultiQC (version 1.4)^30^. Samtools (version 0.0.19)^31^ and IGV (version 2.6.2)^32^ were used for indexing and viewing the alignments, respectively. Gene expression was quantified as gene level counts using the htseq-count function (version 0.12.3)^33^; the Ensembl gene annotations for the human genome were used (genebuild-last-updated 2019-06). The htseq-count default parameters were used. Differentially expressed genes were quantified using DESeq2 (version 1.28)^34^ on R platform (Version 4.0.1) (https://www.r-project.org/). DESeq2 was run with the default parameters^34^. The normalized gene expression data were used for the downstream analyses such as sorting and data visualization (heat maps, boxplots, and ladder plots). The 13 Illumina RNA-seq samples have been reported and described before (accession code of GSE148158)^9,10^.

### Human transcription factors

Human transcript factors were based on the revised version of Lambert et al.^17^. *DUX1* and *DUX3* were missing from our RNA-seq data set because they are not annotated in Ensembl or UCSC database. The official symbol for *ZNF645* used in the Lambert list is *CBLL2*. The official symbol for *T* used in the Lambert list is *TBXT*. *ZUFSP* is *ZUP1*, and *ZZZ3* is *AC118549.1*. *ZNF788* (*ZNF788P*) was excluded from analyses since it is annotated in Ensembl and Genecards as a pseudogene. As a result, this research evaluated 1636 human TFs out of the 1639 TFs in the Lambert list.

### Criteria for an active gene with similar expression in both cell types

A gene is considered active in both cell types with equal expression when the following conditions are met: (1) all replicates have a normalized read count greater than 50; (2) the q value should be greater than 0.01; (3) the fold difference between the two cell types should not be equal or greater than 2 regardless of the q value. In this group, all normalized individual read counts are > 50.

### Defining the inactive gene set for both cell types

All normalized read counts are < 50 including all replicates for both cell types. The rationale of this value as the threshold of an active gene has been described before^9^.

### Defining activatome (hPSC-specific gene set) and erasome (fibroblast-specific gene set)

A gene will be a member of activatome or erasome when the following conditions are met: (1) the active cell type, e.g. hPSCs for activatome, should have a normalized read count of greater 50 for all individual replicates while the silent cell type should have a normalized read count less than 50 for all individual replicates; (2) the fold differences should be greater than 2; (3) the q value should be less than 0.01.

### Defining upreprogramome (hPSC-enriched gene set) and downreprogramome (fibroblast-enriched gene set)

An enriched gene in any cell type should meet the following criteria: (1) the normalized read count should be greater than 50 for all replicates; (2) the enrichment should be at least 2-fold; (3) the q value should be < 0.01. The rationale for these criteria have been described before^10^.

### Defining the irresponsive TF genes to OSKM induction

For TF downreprogramome and upreprogramome, an irresponsive TF gene to OSKM induction should meet the following criteria: (1) the fold changes should be less than 2-fold (upregulation or downregulation), and any gene with a fold change of > 2-fold is removed from this list regardless of the significant levels; (2) any gene with a significant level of q < 0.01 is removed from the list regardless of the levels of fold changes. i.e., even though the fold change is 1.5-fold it will be removed from the list of irresponsive TF genes if the change is significant; (3) exclude any gene for which the difference between OSKM reprogramming cells and the ESC become less than 2-fold regardless of the significance status.

For upreprogramome, the following additional criteria were applied. If the gene remain inactive after OSKM induction (normalized read count < 50), it is considered irresponsive even though the fold change is > 2 and is statistically significant.

### Data visualization

Heat maps were prepared using the R package *pheatmap* (Version 1.0.12) in RStudio (Version 1.3.1073) (https://rstudio.com/) on a desktop iMAC (Version 10.15.6) as described^35^. Boxplots were generated using the generic R function of *boxplot()* in RStudio as described recently^36^. Both heat maps and boxplots were prepared using the log2-transformed read counts. Ladder plots were generated using the R package of *plotrix* (Version 3.7-8) in RStudio.

## Supplementary information

Supplementary Information 1.

Supplementary Table S1.

Supplementary Table S2.

Supplementary Table S3.

Supplementary Table S4.

Supplementary Table S5.

Supplementary Table S6.

Supplementary Table S7.

Supplementary Table S8.

Supplementary Table S9.

Supplementary Table S10.

Supplementary Table S11.

Supplementary Table S12.

Supplementary Table S13.

Supplementary Table S14.

Supplementary Table S15.

Supplementary Table S16.

Supplementary Table S17.

## Data Availability

*Accession numbers* The RNA-seq data have been deposited in Gene Expression Omnibus (GEO) with the accession code of GSE148158, and GSE159410.
