# Supplementary material for: Human transcription factors responsive to initial reprogramming predominantly undergo legitimate reprogramming during fibroblast conversion to iPSCs
Source: Sci Rep. 2020 Nov 12;10:19710. doi: 10.1038/s41598-020-76705-y (PMC7661723; doi:10.1038/s41598-020-76705-y)
Supplement: Supplementary file 1 — Supplementary Information 1. [file 41598_2020_76705_MOESM1_ESM.docx]

**Supplemental Information**

**Human transcription factors responsive to initial reprogramming predominantly undergo legitimate reprogramming during fibroblast conversion to iPSCs**

Ricardo Cevallos ^1^, Yvonne J. K. Edwards ^1,2^, John M Parant ^3^, Bradley K Yoder ^2^, Kejin Hu^1,^ *

Department of Biochemistry and Molecular Genetics ^1^, Department of Cell Developmental and Integrative Biology ^2^, Department of Pharmacology and Toxicology ^3^, School of Medicine, University of Alabama at Birmingham, Birmingham, AL, USA, 35294

*Correspondence and lead contact: [kejinhu@uab.edu](mailto:kejinhu@uab.edu)

Figure S1. 279 transcription factors are enriched by at least 2-fold in fibroblast compared to human ESCs.

1. A heat map showing higher expression of 279 transcription factors in fibroblasts than in human ESCs.
2. Box plots showing overall higher expression of the 279 human TFs in each of the fibroblast samples than in ESCs.
3. Ladder plots showing individually higher expression of the 279 TF genes in human fibroblasts than in ESCs.

ESC = 3; fibroblasts = 4; q < 0.01. ESC samples are highlighted in green. H1 and H9, human embryonic stem cell lines H1 and H9, respectively. BJ, human fibroblasts BJ cells. Number after BJ is the hours after seeding at the time of RNA extraction. The last number of the fibroblast designation is the lane number of RNA-seq flow cells.

Figure S2. 310 transcription factors are enriched by at least 2-fold in human ESCs compared to fibroblasts.

1. A heat map showing higher expression of 310 transcription factors in human ESCs than in fibroblasts.
2. Box plots showing overall higher expression of the 310 human TFs in in ESCs than in fibroblasts.
3. Ladder plots showing individually higher expression of the 301 TF genes in human ESCs than in fibroblasts.

ESC = 3; fibroblasts = 4; q < 0.01. ESC samples are highlighted in green. H1 and H9, human embryonic stem cell lines H1 and H9, respectively. BJ, human fibroblasts BJ cells. Number after BJ is the hours after seeding at the time of RNA extraction. The last number of the fibroblast designation is the lane number of RNA-seq flow cells.

Figure S3. List of the unique GO terms to PSCs and associated data for the GO analyses of “biological process complete” for the ESC-enriched genes. FDR < 0.01.

Figure S4. List of the representative unique GO terms to fibroblasts and associated data for the GO analyses of “biological process complete” for the fibroblast-enriched genes. FDR < 0.01.

Figure S5. 108 fibroblast-enriched TFs were resistant to OSKM reprogramming at early stages.

(A) Box plots showing that the 108 fibroblast-enriched TF as a group remain similar expression levels as that of the naïve fibroblasts and fibroblasts transduced with GFP control viruses.

(B) Ladder plots showing that the 108 fibroblast-enriched TFs remain individually similar expression levels as that of the naïve fibroblasts and fibroblasts transduced with GFP control viruses.

Figure S6. 188 PSC-enriched TFs were resistant to OSKM reprogramming at early stages.

(A) Box plots showing that the 188 PSC-enriched TF as a group remain similar expression levels as that of the naïve fibroblasts and fibroblasts transduced with GFP control viruses.

(B) Ladder plots showing that the 188 PSC-enriched TFs remain individually similar expression levels as that of the naïve fibroblasts and fibroblasts transduced with GFP control viruses.

Figure S7. 49 transcription factors were significantly upregulated by OSKM expression 48 hours post OSKM transduction.

(A) A heat map showing upregulation of 49 transcription factors by OSKM (highlighted in red). Please note that OCT4, SOX2, KLF4 and MYC were included in the heat map as well because of the sorting criteria, but the upregulation is due to transgene overexpression.

(B) Box plots showing upregulation of 49 TF genes by OSKM (highlighted in red) at the early stages of iPSC reprogramming.

(C) Ladder plots showing individually upregulation of the 49 TFs by OSKM induction (highlighted in red).

Figure S8. 70 transcription factors were significantly downregulated by OSKM overexpression 48 hours post OSKM transduction.

(A) A heat map showing downregulation of 70 transcription factors by OSKM (highlighted in red).

(B) Box plots showing downregulation of 70 TF genes by OSKM at the early stages of iPSC reprogramming.

(C) Ladder plots showing individually downregulation of the 70 TFs by OSKM (highlighted in red).

Figure S9. 21 OSKM-downregulated TFs were properly reprogrammed at the initial stages of iPSC reprogramming.

(A) Box plots showing successful downreprogramming of 21 TFs.

(B) Ladder plots showing successful downreprogramming of 21 TFs.

Figure S10. 33 fibroblast-enriched and OSKM-downregulated TFs were legitimately but insufficiently reprogrammed at the initial stages of iPSC reprogramming.

(A) Box plots showing significant but insufficient downreprogramming of 33 TFs.

(B) Ladder plots showing significant but insufficient downreprogramming of 33 fibroblast-enriched TFs.

Figure S11. 18 ESC-enriched TFs upregulated by OSKM were properly upreprogrammed at the initial stages.

1. Box plots showing that the 18 ESC-enriched TFs have been upreprogrammed to similar expression levels of to that in ESCs.

(B) Ladder plots showing that the 18 ESC-enriched TFs have been upreprogrammed to similar expression levels of to that in ESCs.

Figure S12. 11 fibroblast-enriched TFs significantly upregulated by OSKM were insufficiently upreprogrammed at the initial stages.

(A) Box plots showing overall insufficient upreprogramming of the 11 fibroblast-enriched TFs to that in ESCs.

(B) Ladder plots showing individually insufficient upreprogramming of the 11 fibroblast-enriched TFs to that in ESCs.

Figure S13. The two different human fibroblasts (CRL and BJ) share very similar TF up-reprogramomes. A, Venn diagram showing numbers of transcription factors enriched in human ESCs compared to the two types of human fibroblasts (CRL and BJ). B, A heat map showing significant higher expression of the 263 TF in human ESCs than in both fibroblasts. C, 59 of the 87 TF, that is enriched in ESC compared to CRL cells (q < 0.01, >2×), are still expressed at significantly higher levels in human ESCs than in BJ cells when the sorting criteria were loosened (p < 0.05 at any fold change rather than >2×, without restriction of a minimum read count). D, 28 of the 47 TF, that is enriched in ESCs when compared to BJ (q < 0.01, >2×), are still expressed at significantly higher levels in human ESCs than in CRL cells when the sorting criteria were loosened (p < 0.05 at any fold change rather than >2×, without restriction of a minimum read count). Samples of ESC are indicated with red color.

Figure S14. The two different human fibroblasts (CRL and BJ) share very similar TF down-reprogramomes. A, Venn diagram showing numbers of transcription factors enriched in human fibroblasts (CRL and BJ) compared to ESCs. B, A heat map showing significant higher expression of 226 TFs in both fibroblasts (CRL and BJ) than in ESCs. C, 31 of the 53 TFs, that is enriched in CRL cells compared to ESCs (q < 0.01, >2×), are still expressed at significantly higher levels in the other fibroblast BJ than in ESCs when the sorting criteria were loosened (p < 0.05 at any fold change rather than >2×, without restriction of a minimum read count). D, 27 out of the 53 TFs, that is enriched in BJ fibroblast when compared to ESCs (q < 0.01, >2×), are still expressed at significantly higher levels in the other fibroblast CRL than in ESCs when the sorting criteria were loosened (p < 0.05 at any fold change rather than >2×, without restriction of a minimum read count). Samples of ESC are indicated with red color.

Figure S15. The four reprogramming factors (OSKM) were overexpressed well in the reprogramming cells that were RNA-sequenced and used in this study. **A**, Representative images of the transduced human fibroblasts with OSKM reprogramming factors, which co-express GFP. Left, brightfield; middle, GFP; right, merged. Please note that after merging some cells lost GFP signals, especially for those with weak GFP expression. Scale bar, 100 μm. 10× objective. **B**, Log2(fold change) for each of the four reprogramming factors in the four RNA samples sequenced in this study. The four CRL fibroblasts were transduced with the four factors together and RNAs were harvested at 48 or 72 hours post transduction. The fold changes (FC) were calculated by dividing the normalized read counts of the transgene samples by the averaged normalized read counts of the naive CRL fibroblasts. **C**, Log2(FC) for the reprogramming factors in the reprogramming BJ cells at 96 hours post transduction. FC is similarly calculated as in S17A. **D**, Lo2(FC) for the each of the four reprogramming factors in the reprogramming BJ cells at 48 and 72 hours post transduction.

Figure S16. The 129 TFs were legitimately upreprogrammed in both BJ and CRL starting cells at 48 hours, 72 hours and 96 hours as shown by heat map and clustering (A), and boxplot (B). Both heat maps and boxplots were drawn using the log2-transformed normalized read counts.

Figure S17. The 89 TFs were legitimately downreprogrammed in both BJ and CRL starting cells at 48, 72 and 96 hours as shown by heat map and clustering (A), and boxplot (B). Both heat maps and boxplots were drawn using the log2-transformed normalized read counts.

**Table S1. Normalized read counts and the related metadata and statistics for the 1,636 human transcription factors based on RNA-Seq data of human fibroblasts and embryonic stem cells (ESCs).**

**Table S2. Normalized read counts and the related metadata and statistics for the 279 fibroblast-enriched TFs based on RNA-Seq data of human fibroblasts and ESCs.** ESC = 3; fibroblasts = 4. Fold enrichment >2; q < 0.01. Normalized read count for any of the fibroblast-enriched gene is greater than 50 for all samples. The zinc finger TFs are highlighted in red.

**Table S3. Normalized read counts and the related metadata and statistics for the 93 TFs expressed only in human fibroblasts based on RNA-Seq data of human fibroblasts and ESCs.** ESC = 3; fibroblasts = 4. Fold enrichment >2; q < 0.01. Normalized read count for any of the TF gene in fibroblasts is greater than 50 for all of the samples.

**Table S4. Normalized read counts and the related metadata and statistics for the 310 TFs enriched in human ESCs based on RNA-Seq data of human fibroblasts and ESCs.** ESC = 3; fibroblasts = 4. Fold enrichment >2; q < 0.01. Normalized read counts for any of the ESC-enriched gene are greater than 50 for all samples. The zinc finger TFs are highlighted in red.

**Table S5. Normalized read counts and the related metadata and statistics for the 70 TFs expressed only in human ESCs based on RNA-Seq data of human fibroblasts and ESCs.** ESC = 3; fibroblasts = 4. Fold enrichment >2; q < 0.01. Normalized read count for any of the ESC-enriched gene is greater than 50 for all samples. Zinc finger TFs are highlighted in red.

**Table S6. List of overrepresented GO terms in downreprogramome for analysis of “biological process complete”**. FDR < 0.01. Based on PANTHER GO database.

**Table S7. List of overrepresented GO terms in upreprogramome for analysis of “biological process complete”**. FDR < 0.01. Based on PANTHER GO database.

**Table S8. Normalized read counts and the related data for the 108 fibroblast-enriched TFs that were resistant to OSKM reprogramming at the earl stage of OSKM reprogramming.**

**Table S9. Normalized read counts and the related data for the 188 PSC-enriched TFs that were resistant to OSKM reprogramming at the earl stage of OSKM reprogramming.** Zinc finger TFs are highlighted in red.

**Table S10. 49 human TFs were significantly upregulated by OSKM at the early stage of iPSC reprogramming**. q < 0.01. FC > 2 compared to both naïve fibroblast (n=4) and fibroblasts transduced with GFP viruses (n = 4). Please note that the 4 reprogramming factors *POU5F1*, *SOX2*, *KLF4* and *MYC* are also included in this group due to the sorting criteria used.

**Table S11. 70 human TFs were significantly downregulated by OSKM at the early stage of iPSC reprogramming**. q < 0.01. FC > 2 compared to both naïve fibroblast (n = 4) and fibroblasts transduced with GFP viruses (n = 4).

**Table S12. Most of the 70 human TFs downregulated by OSKM is legitimate reprogramming**. (has overlap with supplementary table 11).

**Table S13. Transcript factors with significant higher expression in human ESCs compared to an independent human fibroblast line CRL (>2×, q < 0.01, normalized read counts for all ESC samples > 50)**.

**Table S14. Transcript factors (279 genes) with significant higher expression in human fibroblast CRL than in ESCs (>2×, q < 0.01, normalized read counts for all CRL samples > 50).**

**Table S15. Summary for the 219 transcript factors that were significantly upregulated by OSKM in an independent human fibroblast CRL (>2×, q < 0.01, normalized read counts for all OSKM samples > 50).**

**Table S16. Summary for the 118 transcript factors that were significantly downregulated by OSKM in an independent human fibroblast CRL (>2×, q < 0.01, normalized read counts for all fibroblast samples > 50).**

**Table S17.** **Summaries for the 19 DNB-seq RNA-seq samples.**
