# Supplementary material for: Human transcription factors responsive to initial reprogramming predominantly undergo legitimate reprogramming during fibroblast conversion to iPSCs
Source: Sci Rep. 2020 Nov 12;10:19710. doi: 10.1038/s41598-020-76705-y (PMC7661723; doi:10.1038/s41598-020-76705-y)
Supplement: Supplementary file 18 — Supplementary Table S17. [file 41598_2020_76705_MOESM18_ESM.docx]

Table S17. Summaries for the 19 DNB-seq RNA-seq samples (GEO#, GSE159410).

| Sample | Uniquely mapped | Mapped to multiple loci | Mapped to too many loci | Unmapped: too short | Unmapped: other | Total |
| --- | --- | --- | --- | --- | --- | --- |
| BJ48hS_1 | 25,207,329 | 838,364 | 3,887 | 323,944 | 2,655 | 26,376,179 |
| BJ48hS_2 | 27,402,260 | 872,339 | 5,731 | 315,681 | 5,688 | 28,601,699 |
| BJ48HS_3 | 29,631,699 | 851,941 | 3,756 | 354,948 | 3,087 | 30,845,431 |
| BJ72HS_3 | 29,498,962 | 843,358 | 3,264 | 390,810 | 3,077 | 30,739,471 |
| BJOSKM96HS_1 | 28,983,709 | 1,033,966 | 9,213 | 1,038,221 | 6,217 | 31,071,326 |
| BJOSKM96HS_2 | 29,605,416 | 981,774 | 8,160 | 938,924 | 6,302 | 31,540,576 |
| BJOSKM96HS_3 | 29,654,380 | 906,703 | 8,550 | 893,526 | 6,292 | 31,469,451 |
| CRL48HS_1 | 30,487,905 | 790,089 | 3,814 | 514,178 | 3,174 | 31,799,160 |
| CRL48HS_2 | 30,603,968 | 807,996 | 4,330 | 417,621 | 3,188 | 31,837,103 |
| CRL72HS_1 | 29,558,225 | 746,004 | 3,763 | 504,895 | 3,079 | 30,815,966 |
| CRL72HS_2 | 29,644,948 | 740,449 | 3,930 | 413,066 | 3,083 | 30,805,476 |
| CRLOSKM48HS_1 | 29,752,479 | 915,849 | 6,228 | 1,201,899 | 3,188 | 31,879,643 |
| CRLOSKM48HS_2 | 29,753,805 | 920,313 | 6,784 | 1,125,080 | 3,187 | 31,809,169 |
| CRLOSKM72HS_1 | 28,934,353 | 1,260,591 | 10,836 | 1,702,825 | 6,378 | 31,914,983 |
| CRLOSKM72HS_2 | 28,482,878 | 1,085,717 | 9,350 | 1,208,519 | 6,150 | 30,792,614 |
| H1P4_4 | 29,421,526 | 1,064,150 | 6,945 | 398,135 | 6,173 | 30,896,929 |
| H9P69S2_3 | 29,196,231 | 1,085,798 | 10,722 | 537,740 | 6,181 | 30,836,672 |
| hESH_1 | 27,155,345 | 1,151,081 | 18,144 | 319,794 | 5,762 | 28,650,126 |
| hESH_2 | 25,877,560 | 1,227,429 | 28,998 | 331,974 | 8,231 | 27,474,192 |
